# Efficacy of dexmedetomidine on myocardial ischemia/reperfusion injury in patients undergoing cardiac surgery with cardiopulmonary bypass: A protocol for systematic review and meta-analysis

**DOI:** 10.1097/MD.0000000000033025

**Published:** 2023-03-03

**Authors:** Gencheng Liang, Yueyong Li, Sheng Li, Zhaohe Huang

**Affiliations:** a Department of Emergency, Affiliated Hospital of Youjiang Medical University for Nationalities, Guangxi, China; b Department of Intervention Medicine, Affiliated Hospital of Youjiang Medical University for Nationalities, Guangxi, China; c Graduate School, Youjiang Medical University for Nationalities, Guangxi, China.

**Keywords:** cardiac surgery, cardiopulmonary bypass, dexmedetomidine, ischemia, reperfusion

## Abstract

Cardiac surgery using cardiopulmonary bypass has been shown to cause reversible postischemic cardiac dysfunction and is associated with reperfusion injury and myocardial cell death. Therefore, it is very important to have a series of measures in place to reduce oxygen consumption and provide myocardial protection. We performed a protocol for systematic review and meta-analysis to evaluate the effect of dexmedetomidine administration on myocardial ischemia/reperfusion injury in patients undergoing cardiac surgery with cardiopulmonary bypass.

**Methods:**

This review protocol is registered in the PROSPERO International Prospective Register of systematic reviews, registration number CRD42023386749. A literature search is performed in January 2023 without restriction to regions, publication types or languages. The primary sources were the electronic databases of PubMed, Embase, Web of Science, the Cochrane Central Register of Controlled Trials, Chinese National Knowledge Infrastructure database, Chinese Biomedical Database, and Chinese Science and Technology Periodical database. Risk of bias will be assessed according to the Cochrane Risk of Bias Tool. The meta-analysis is performed using Reviewer Manager 5.4.

**Results:**

The results of this meta-analysis will be submitted to a peer-reviewed journal for publication.

**Conclusion:**

This meta-analysis will evaluate the efficacy and safety of dexmedetomidine in patients undergoing cardiac surgery with cardiopulmonary bypass.

## 1. Introduction

Cardiopulmonary bypass (CPB) provides blood and oxygen to the body’s organs and tissues when the heart stops working, allowing open-heart surgery to be performed successfully and maintaining the body’s metabolism.^[1–3]^ However, owing to the lack of blood supply to the heart during CPB, perioperative myocardial protection, which directly affects the recovery of cardiac function postsurgery, is very important.^[4,5]^ Myocardial protection strategy refers to a variety of perioperative techniques used to reduce ischemia–reperfusion injury and prevent postoperative cardiac dysfunction.

The main method is to reduce myocardial oxygen demand, such as inducing lower body temperature and nonbeating heart. In addition to myocardial injury, the inflammatory response related to CPB is also 1 of the issues worthy of our concern in open-heart surgery. CPB provokes a vigorous systemic inflammatory response, induced by the exposure of blood elements to nonphysiological surfaces, resulting in myocardial damage.^[4]^ Furthermore, excluding the heart from the systemic circulation renders the myocardium ischemic and upon reperfusion, and triggers postischaemic myocardial dysfunction. Central in the pathogenesis of ischemic myocardial injury is the depletion of high-energy phosphates and the disturbance of normal intracellular calcium homeostasis.

Anesthetic agents can exert clinical benefits during and after surgery. Optimistically, the ideal antiinflammatory anesthetic should reduce complications and mortality resulting from systemic inflammatory response syndrome-like postoperative responses. Alpha 2-adrenergic receptor agonists have been used in anesthesia because of their sedative, analgesic, hemodynamic-stabilizing, and sympatholytic effects. In addition, the stress response to surgery can be modulated by postsynaptic central α2-adrenergic receptor activation. In this regard, dexmedetomidine is considered as a promising candidate.^[6–9]^ However, only a few studies have investigated the efficacy and safety of dexmedetomidine after cardiac surgery with CPB. Therefore, we performed a protocol for systematic review and meta-analysis to evaluate the effect of dexmedetomidine administration on myocardial ischemia/reperfusion injury in patients undergoing cardiac surgery with CPB.

## 2. Methods

### 2.1. Study registration

This review protocol is registered in the PROSPERO International Prospective Register of systematic reviews, registration number CRD42023386749. It has been reported following the preferred reporting items for systematic reviews and meta-analyses protocol.^[10]^ All studies included in this meta-analysis come from public research databases. Ethical approval is not required for this study.

### 2.2. Inclusion and exclusion criteria

Inclusion criteria: Participants: Patients more than 18 years old undergoing cardiovascular surgery with CPB; Interventions: The intervention of the experimental group received intravenous dexmedetomidine; Comparison: control group received same amount of normal saline or matched placebo; Outcomes measures: creatinine kinase-MB concentration, cTn-I concentration, and the length of a patient’s ICU stay; Study design: Only randomized controlled trials will be included.

The exclusion criteria were as follows: the study lacked key information or usable data; the articles were review articles, letters, single case reports, or conference abstracts. In cases of multiple articles from the same group that reported overlapping data, only the most comprehensive articles were included.

### 2.3. Search strategy

A literature search is performed in January 2023 without restriction to regions, publication types or languages. The primary sources are the electronic databases of PubMed, Embase, Web of Science, the Cochrane Central Register of Controlled Trials, Chinese National Knowledge Infrastructure database, Chinese Biomedical Database, and Chinese Science and Technology Periodical database. Two authors will independently draft and carry out the search strategy. The gray literature will be searched in databases. Articles will also be searched from the references of the retrieved studies. The key terms used for the search are “dexmedetomidine,” “myocardial ischemia,” “reperfusion injury,” “cardiac surgery,” and “cardiopulmonary bypass” separately and in combination.

### 2.4. Study selection

**Figure 1.**
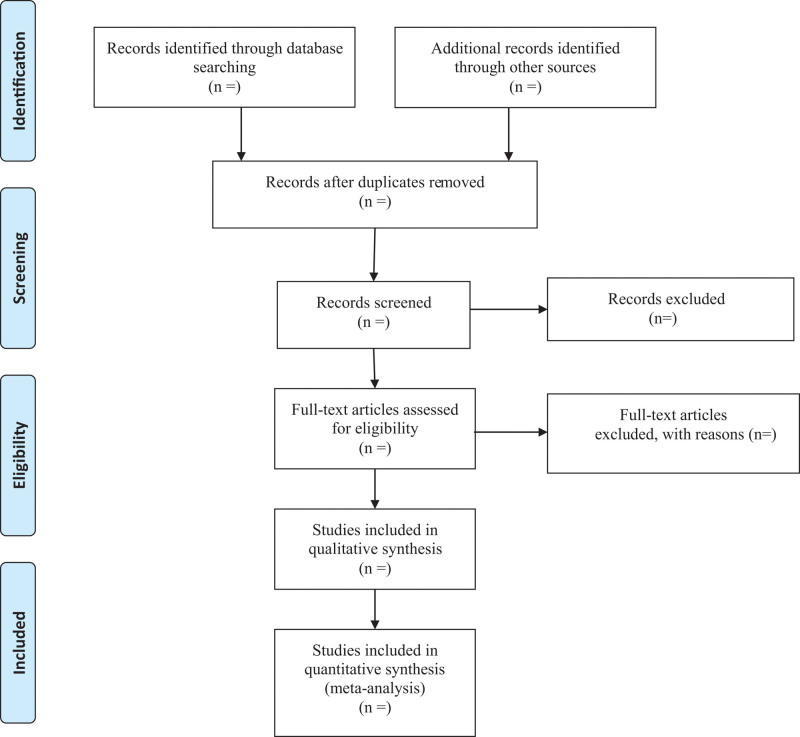
Flow diagram of study selection process.

Study selection will be carried out by 2 independent experienced researchers. Endnote X9 acts as a literature management tool for retrieved articles. After the software automatically removes duplicate references, they will screen preliminarily the title and abstract to determine whether inclusion criteria are met. Subsequently the selected literature will be downloaded in full text for more detailed screening. In case of controversy, a third investigator will join the discussion. All of the excluded articles will be marked with reasons. The whole selection process will be presented in a PRISMA flow diagram (Fig. 1).

### 2.5. Data extraction.

The following information will be collected by a predetermined data form generated by Microsoft Excel: basic information (title, first author, year of publication, type of study, and location of study); study population (age, sex, sample size, detailed description of participants, and diseases); details of interventions and comparison; related outcomes mentioned above and the length of follow-up time. Any disagreement in this process will be solved by consensus or consultation with a third person.

### 2.6. Risk of bias

Risk of bias will be assessed according to the Cochrane Risk of Bias Tool^[11]^ which bases on the following domains: random sequence generation, allocation concealment, blinding of participants and personnel, blinding of outcome assessment, incomplete outcome data, selective outcome reporting, and other sources of bias. Items are scored as low, high, or unclear risk of bias. Two independent researchers will attend the evaluation which will be cross-checked by a third senior 1.

### 2.7. Data synthesis

The meta-analysis is performed using Reviewer Manager 5.4. The 95% confidence intervals are used, mean differences are calculated for continuous variables, and risk ratios are calculated for dichotomous variables. Data heterogeneity is assessed using the chi-square and *I*^2^ tests. When heterogeneity is not significant (*P* ≥ .1, *I*^2^ ≤ 50%), a fixed-effects model is used for analysis; when heterogeneity is significant (*I*^2^ > 50% or *P* < .1), a random-effects model is used. In cases of significant heterogeneity among the included studies, we perform a subgroup analysis. This will be explored according to age, gender, race, treatment period, sample size, disease category, and other factors that may affect the results.

### 2.8. Grading quality of evidence

We will use the grading of recommendations assessment, development, and evaluation to assess the results.^[12]^ In the grading of recommendations assessment, development, evaluation system, and the quality of evidence will be categorized into 4 levels: high, moderate, low, and very low quality.

## 3. Discussion

Cardiac surgery using CPB has been shown to cause reversible postischemic cardiac dysfunction and is associated with reperfusion injury and myocardial cell death.^[13,14]^ Therefore, it is very important to have a series of measures in place to reduce oxygen consumption and provide myocardial protection. As a highly selective α2-adrenergic receptor agonist, dexmedetomidine can reduce the release of cytokines, inhibit inflammation response and alleviate ischemia–reperfusion injury, thus exerting its organ-protective effects.^[15,16]^ Changes in kinase-MB concentration, cTn-I serum concentrations may provide a more precise diagnostic evaluation as to the extent of a patient’s myocardial injury.^[17]^ This is the first meta-analysis to systematically evaluate the cardioprotective effect of dexmedetomidine on cardiac surgery under CPB and its effect on accompanied inflammation. However, further studies are needed to explore the effect of dexmedetomidine on the long-term prognosis of patients.

## Author contributions

**Conceptualization:** Yueyong Li.

**Investigation:** Sheng Li.

**Writing – original draft:** Gencheng Liang.

**Writing – review & editing:** Zhaohe Huang.
